# 3-D joint space mapping at the ankle from weight-bearing CT: reproducibility, repeatability, and challenges for standardisation

**DOI:** 10.1007/s00330-023-09718-6

**Published:** 2023-05-31

**Authors:** Tom D. Turmezei, Karan Malhotra, James W. MacKay, Andrew H. Gee, Graham M. Treece, Kenneth E. S. Poole, Matthew J. Welck

**Affiliations:** 1 Department of Radiology, Norfolk and Norwich University Hospital NHS Foundation Trust, Colney Lane, Norwich, UK; 2 Norwich Medical School, University of East Anglia, Norwich Research Park, Norwich, UK; 3 Royal National Orthopaedic Hospital NHS Trust, Brockley Hill, Stanmore, UK; 4 Department of Radiology, University of Cambridge, Hills Road, Cambridge, UK; 5 Cambridge University Engineering Department, Trumpington Street, Cambridge, UK; 6 Department of Medicine, University of Cambridge, Hills Road, Cambridge, UK

**Keywords:** Cone-beam computed tomography, Ankle joint, Weight-bearing, Osteoarthritis, Reproducibility of results

## Abstract

**Objectives:**

We present a 3-D approach to joint space width (JSW) measurement across the ankle from weight-bearing CT (WBCT) to demonstrate inter-operator reproducibility, test-retest repeatability, and how differences in angulation affect ankle JSW distribution.

**Methods:**

One side from repeat WBCT imaging of both feet and ankles was analysed from 23 individuals as part of their routine clinical care pathway. Joint space mapping was performed at four facets across the talus: talonavicular, talar dome and medial gutter (dome-medial), lateral gutter, and posterior subtalar. Inter-operator reproducibility was calculated for two users, while test-retest repeatability was calculated by comparing the two visits, both presented as Bland-Altman statistics. Statistical parametric mapping determined any significant relationships between talocrural joint space angulation and 3-D JSW distribution.

**Results:**

The average ± standard deviation interval between imaging was 74.0 ± 29.6 days. Surface averaged bias ± limits of agreement were similar for reproducibility and repeatability, the latter being: talonavicular 0.01 ± 0.26 mm, dome-medial 0.00 ± 0.28 mm, lateral gutter − 0.02 ± 0.40 mm, and posterior subtalar 0.02 ± 0.34 mm. Results are presented as 3-D distribution maps, with optimum test–retest repeatability reaching a smallest detectable difference of ± 0.15 mm.

**Conclusions:**

Joint space mapping is a robust approach to 3-D quantification of JSW measurement, inter-operator reproducibility, and test–retest repeatability at the ankle, with sensitivity reaching a best value of ± 0.15 mm. Standardised imaging protocols and optimised metal artefact reduction will be needed to further understand the clinical value of these 3-D measures derived from WBCT.

**Clinical relevance statement:**

Weight-bearing computed tomography is an increasingly important tool in the clinical assessment of orthopaedic ankle disorders. This paper establishes the performance of measuring 3-D joint space width using this technology, which is an important surrogate marker for severity of osteoarthritis.

**Key Points:**

• *Joint space width values and error metrics from across the ankle measured from weight-bearing CT can be presented as 3-D maps that show topographic variation*.

• *The best sensitivity for detecting meaningful change in 3-D joint space width at the ankle was* ± *0.15 mm, a value less than the isotropic imaging voxel dimensions*.

• *Standardised imaging protocols and optimised metal artefact reduction will be needed to understand the clinical value of 3-D measures from weight-bearing CT*.

**Supplementary information:**

The online version contains supplementary material available at 10.1007/s00330-023-09718-6.

## Introduction

Since the first reports of cone-beam CT technology in orthopaedic imaging just over a decade ago [1], weight-bearing computed tomography (WBCT) has played an increasingly important role in the imaging of foot and ankle disorders [2, 3]. Early clinical adopters have used WBCT for assessing degenerative joint disease, distal tibiofibular syndesmosis injury, complex foot deformity, joint alignment, and for planning arthroplasty [4–6]. This has been under the premise that imaging in a weight-bearing position provides a more realistic representation of the foot and ankle, particularly when considering joint space width (JSW) and bone alignment.

Assessment of JSW with WBCT has mostly focused on single-value measurements made at selected locations or in pre-set imaging planes, but some studies have delivered software-driven 3-D geometric measurements of the distance between bone surfaces [4, 7]. Various techniques have been developed for the measurement of JSW in 3-D, usually the distance between segmented bone surfaces with values reported as a mean from within pre-defined subregions [8, 9]. However, such an approach may be sub-optimal because JSW accuracy will depend on segmentation performance. Furthermore, JSW values at the foot and ankle may be blurred by the resolving capabilities of the imaging system, a problem exacerbated by reliance on segmentation of bone surfaces to provide these measures [10].

The inter-operator reproducibility and test-retest repeatability of JSW measurement techniques are also rarely reported in a way that quantitatively evaluates performance, let alone in 3-D [11–13]. However, without quantification, performance cannot be objectively evaluated, which then has consequences for research and clinical applications [14].

In this study, we present a semi-automated 3-D JSW measurement approach called joint space mapping (JSM) that has been previously applied at the hip and knee [12, 15]. Our specific goals were to demonstrate the feasibility of JSM for the first time at the ankle using WBCT imaging in a standard clinical population and to quantify its inter-operator reproducibility and test-retest repeatability in 3-D.

## Materials and methods

### Study participants

This was a retrospective study testing the feasibility and reliability of 3-D JSW assessment from WBCT in a standard clinical population. The Royal National Orthopaedic Hospital, Stanmore, UK, maintains a prospective database of patients who undergo WBCT imaging and this database was interrogated to find suitable patients scanned in the designated study period between 2013 and 2017. We included adult patients who had two clinically indicated WBCT scans of both feet and ankles performed within 4 months of each other to allow for repeatability testing under the assumption of no progression in joint space narrowing between imaging time points. All scans were performed as part of their regular clinical care pathway. Cases were selected with no prior constraint on joint positioning and required no surgical intervention being performed on either side between scans. There were no constraints on the indications for scanning as inclusion or exclusion criteria so that a mixed clinical population could be included to optimise robustness of reproducibility and repeatability testing. Both feet and ankles were scanned simultaneously in the weight-bearing position as per the institution’s standard imaging protocol. Thirty sequential cases were initially selected that met these criteria as a widely recognised basis for reproducibility and repeatability analysis. The opposite side to the primary indication for scanning was selected for the study (see Table 1), noting subsequent exclusions on technical grounds (below). This selection approach (along with the 4-month interval limit) was the best available to reinforce our assumption that there would be no clinical progression in joint space narrowing between imaging time points.

**Table 1:** Condition and diagnoses in the original 30 participants as the indication for WBCT scanning, noting that the opposite side was selected for inclusion in the study

Indications for imaging on contralateral side	*n*
Forefoot/midfoot fusion delayed union	7
Hindfoot fusion delayed union	5
Ankle fracture delayed union	5
Hallux valgus correction monitoring	4
Forefoot fracture delayed union	4
Monitoring foot deformity correction	2
Monitoring post-surgical deformity	1
Charcot foot	1
No notes available	1

As the site of imaging acquisition, The Royal National Orthopaedic Hospital exercised discretionary power as a public authority under the UK GDPR (General Data Protection Regulations) ‘public task’ Article 6(1)(e) to process information from patients with waiver of the requirements for consent or ethics approval given for this study. A service level agreement between The Royal National Orthopaedic Hospital and The Norfolk and Norwich University Hospital, Norwich, UK, allowed transfer of fully anonymised imaging and demographic data for analysis in Norwich.

### Imaging

All imaging was acquired with cone-beam technology using a CurveBeam (now CurveBeam AI) pedCAT scanner, 120 kVp, 0.37 mm isotropic voxel reconstruction, field of view 35 cm diameter × 20 cm height, with a standard sharp kernel. Acquisition time was approximately 48 s, both sides being imaged at once as the participant stood in the horizontal bore. The dose metrics for each examination were CDTIvol 1.1 mGy, scan length 19.7 cm, and a dose length product (DLP) of 21.7 mGy*cm.

**Fig. 1:**
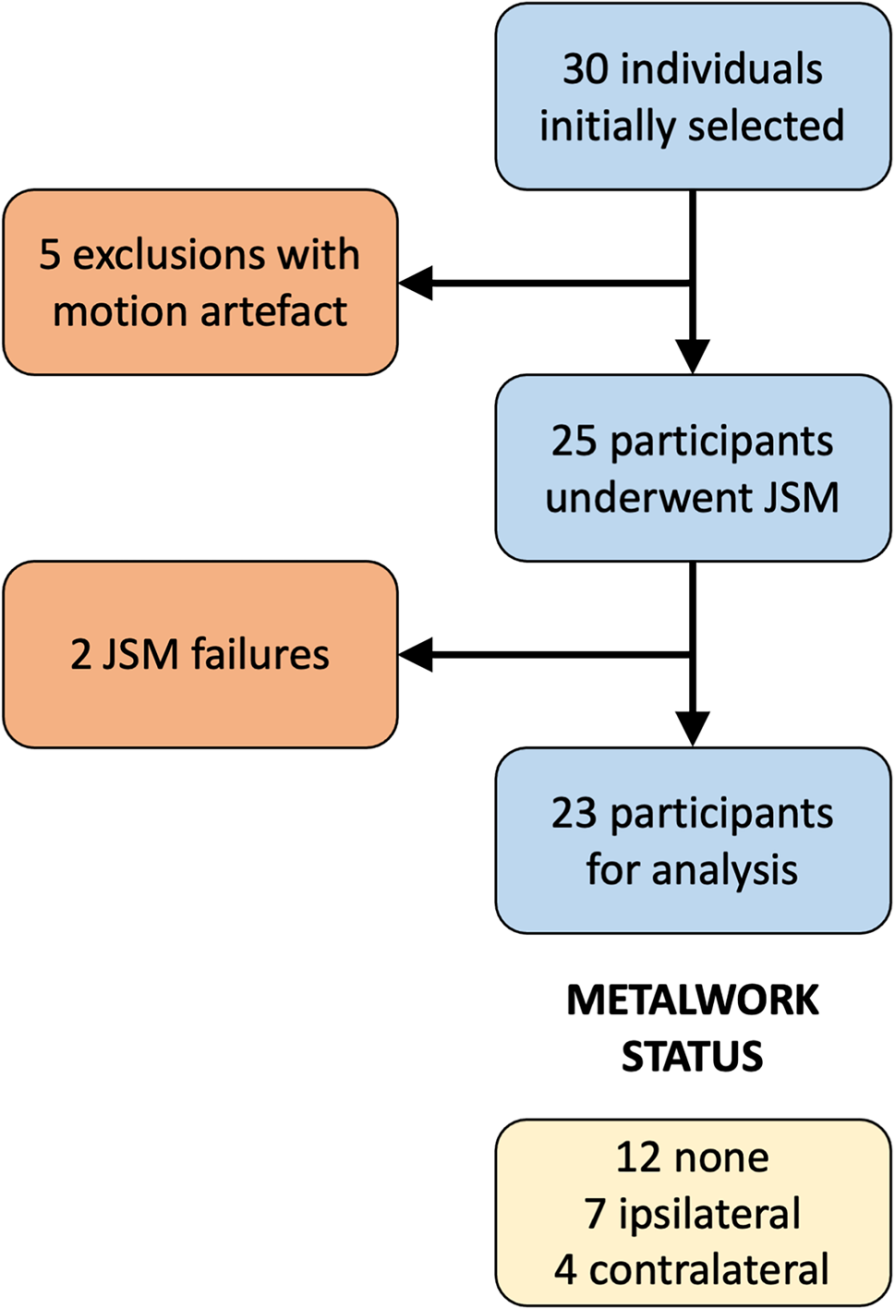
Flow chart of participants from the initial selection of 30 cases to final analysis of 23

**Fig. 2:**
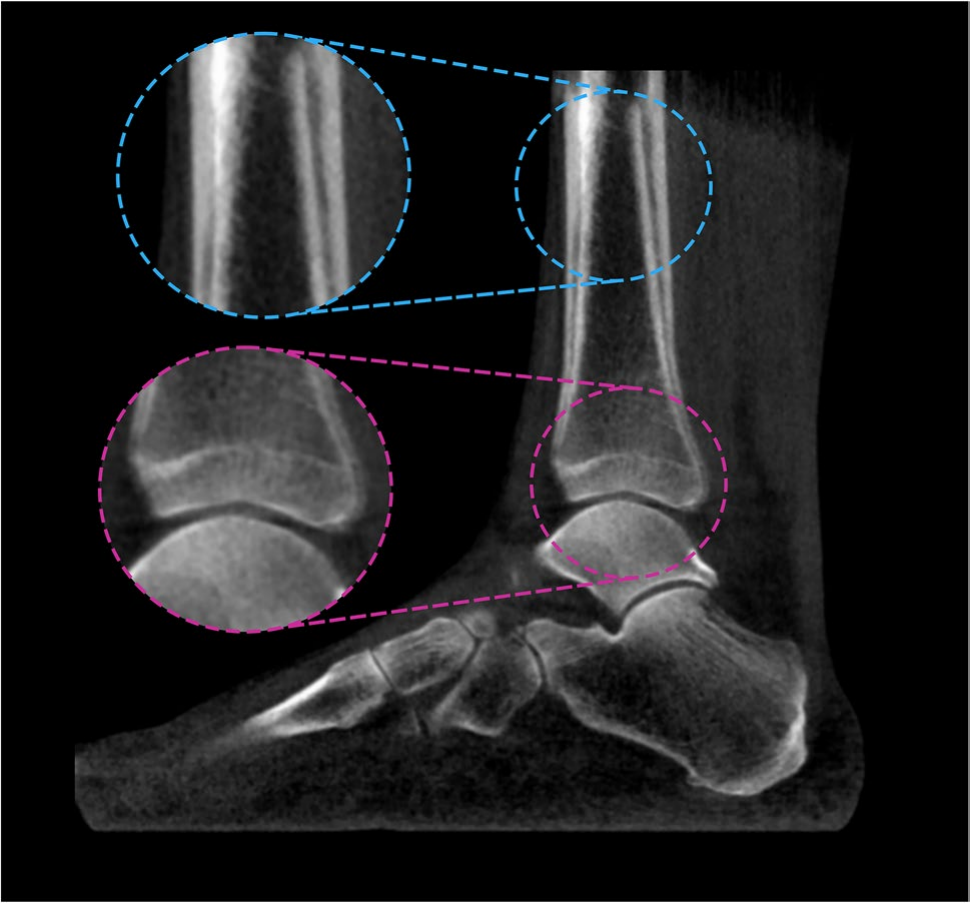
Left ankle of a 64-year-old female with right 1st metatarsophalangeal joint fusion. This case was excluded because of visible motion artefact in the follow-up imaging, as identified at the tibial diaphysis (blue) and more subtly at the tibial plafond (pink)

Five out of the 30 (16.6%) participants were excluded prior to performing JSM because of visible motion artefact in baseline (visit 1) or follow-up (visit 2) imaging (Figs. 1 and 2). Sides for analysis were selected to provide a mix of normal appearances and pathology as judged by one of the study authors (TT), a consultant musculoskeletal radiologist with 10 years of specialist experience.

### Joint space mapping

**Fig. 3:**
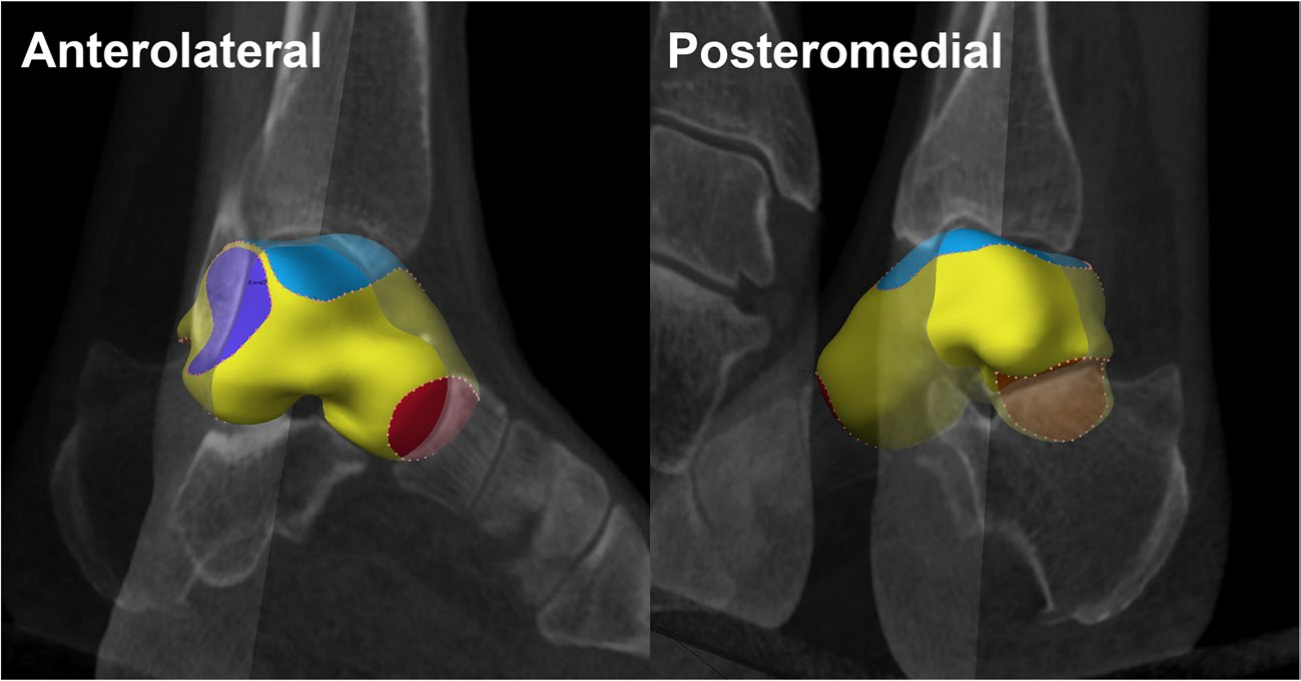
The four joint space patches extracted from the talar surface: talar dome and medial gutter (dome-medial, blue), lateral gutter (purple), talonavicular (red), and posterior subtalar (orange). Each joint space patch is defined as the perimeter to the ‘shadow’ cast from the opposing bone onto the talar surface, manually segmented and then automatically extracted from the surface to act as the framework for subsequent joint space measurement

Given the similarity of JSW magnitude at the ankle compared to the hip, an optimised blur model for JSM was applied in this study [10]. In summary, this involves the following steps that have been previously published at the hip and knee [12, 15]: (1) shape-model-assisted semi-automatic segmentation of the talus from the axial imaging data (~ 20 min per talus); (2) automatic 3-D talar bone mesh surface creation (instantaneous); (3) automatic casting of the ‘shadow’ of opposing bone back onto the talar surface (instantaneous); (4) manual segmentation around the perimeter of the ‘shadow’ for the four joint space patches (~ 5 min per talus); (5) performing JSM automatically across each patch (less than 1 min per talus; Fig. 3 and Supplementary Figs. 1, 2, and 3); (6) automated data smoothing and final surface creation for display and analysis (instantaneous).

All talar segmentations and baseline (visit 1) joint space patch extractions were performed by a single experienced user (TT) at the talar dome and medial gutter (dome-medial, blue patch in Fig. 3), lateral gutter (purple), talonavicular (red), and posterior subtalar (orange) articular surfaces using StradView (version 6.1), in-house software freely available to download at https://mi.eng.cam.ac.uk/Main/StradView. JSW measurement (step 5) was then automatically performed across all joint surfaces. Supplementary Fig. 4 demonstrates how JSM differs from mesh-to-mesh JSW measurement techniques by defining actual bony articular surfaces rather than relying on user-segmented mesh representations to set JSW values.

A novice user trained on six opposite ankles not used in the analysis (JM under the guidance of the TT) then segmented joint space patches at each of the 23 baseline ankles. All 23 patch sets (four patches from each talus) were registered to the average (template) joint surface set using wxRegSurf, in-house software freely available to download at https://mi.eng.cam.ac.uk/~ahg/wxRegSurf/. wxRegSurf performed automatic transfer of measurement data from each individual’s surfaces to the template surfaces. The steps of joint surface creation, joint space mapping, and surface registration have been previously reported in detail [10]. Sagittal talocrural joint angulation was measured by a single assessor (TT) at each ankle from 3-D reconstructions using StradView as the angle set by the lines connecting landmarks at the centre of the distal tibial diaphysis, the centre of the talar dome, and the centre of the talar head articular surface.

### Statistical analysis

The paired Student’s *t*-test was used to determine whether mean talocrural joint angle difference between visits differed significantly from zero degrees, i.e. no change in talocrural joint alignment. Baseline (visit 1) maps for the two users were taken to calculate inter-operator reproducibility across the template surfaces, while follow-up (visit 2) minus baseline (visit 1) maps were taken to calculate test-retest repeatability, both as Bland-Altman statistics with bias ± 95% limits of agreement (LOA) as 1.96 × the standard deviation (SD) of the bias. The limits of agreement can be also taken as the smallest detectable difference in a repeatability study. All results are presented as patch-averaged values at each of the four patch surfaces as well as 3-D distribution maps across the template surfaces. The relationship between talocrural joint angulation (as the degree of plantarflexion) and 3-D JSW distribution was demonstrated with an F test implementation of statistical parametric mapping. This approach controls for the risk of false positive results when making multiple statistical comparisons across a surface, with a *p* value threshold of 0.05 set for significance [16, 17].

## Results

**Fig. 4:**
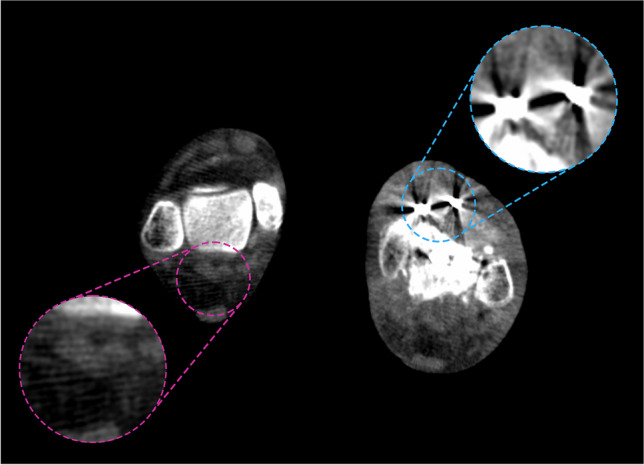
Both ankles of a 76-year-old female participant with left talocrural metalwork fusion. This case had to be excluded because of metal artefact affecting the opposite side with substantial beam hardening as streaks immediately around the fusion metalwork (blue) extending across the frame to the opposite ankle (pink)

Two out of the 25 (8%) final study participants were excluded post hoc because of failure of the JSM density estimation due to the level of metal artefact (Figs. 1 and 4). The average age of the final 23 study participants for analysis was 52.7 years (range 23–74) with 16 females and 7 males, and 13 left side, 10 right side. Twelve ankles had no metalwork, seven had various metalwork fusions in the ipsilateral foot away from the joints of interest, and four had metalwork fusions in the contralateral foot (Fig. 1 and Table 2). The mean ± standard deviation (SD) interval between imaging visits was 74.0 ± 29.6 days. The mean difference in sagittal talocrural joint angulation between visits (visit 2 − visit 1) was not significantly different from zero (mean − 2.7 ± SD 17.7°, *p* = 0.30). Values for individuals are presented in Table 2.

**Table 2:** Breakdown of final 23 participant demographic details, including the side used in the analyses, presence of metalwork at the time of imaging, any prior operation details, the interval between scans, and the difference in angulation of the talocrural joint between visits. Note that it was the opposite side to which imaging was indicated that was included in the analyses

No	Age	Sex	Side	Metalwork	Prior operation details	Interval (d)	Angle Δ*
1	52	Female	Left	-	-	122	− 12.4
2	69	Female	Left	Yes	Left 1^st^ metatarsophalangeal joint fusion	119	− 6.1
3	53	Female	Left	-	-	51	5.2
4	53	Female	Right	Yes	Right 1^st^ tarsometatarsal joint fusion	70	3.4
5	29	Female	Right	-	-	45	− 4.1
6	33	Male	Right	-	-	45	0.9
7	58	Female	Left	Opposite	Right talocrural joint fusion	63	− 7.9
8	49	Female	Right	Yes	Right calcaneal screw fracture fixation	77	5.8
9	73	Female	Right	-		83	13.9
10	62	Male	Right	Opposite	Left talonavicular joint fusion	105	− 5.8
11	72	Male	Left	Yes	Left calcaneal osteotomy and 1^st^ tarsometatarsal joint fusion	121	− 12.7
12	42	Female	Right	-	-	98	17.5
13	67	Female	Left	Opposite	Right anterior subtalar facet and naviculocuneiform joint fusions	88	− 20.4
14	46	Female	Right	-		58	16.2
15	74	Female	Right	Yes	Right tarsometatarsal joint fusions	42	− 5.9
16	43	Male	Right	Yes	Right distal fibular fixation	93	− 1.6
17	48	Female	Left	-	-	108	− 1.4
18	23	Male	Left	-	-	54	2.0
19	52	Male	Left	-	-	42	− 5.7
20	46	Female	Left	Opposite	Right talocrural joint fusion	15	8.9
21	64	Male	Left	-	-	42	− 5.4
22	69	Female	Left	Yes	Right naviculocuneiform joint fusion	92	3.1
23	34	Female	Left	-	-	70	− 2.7

^*^Angle difference as visit 2 − visit 1 in degrees

### Joint space mapping

**Fig. 5:**
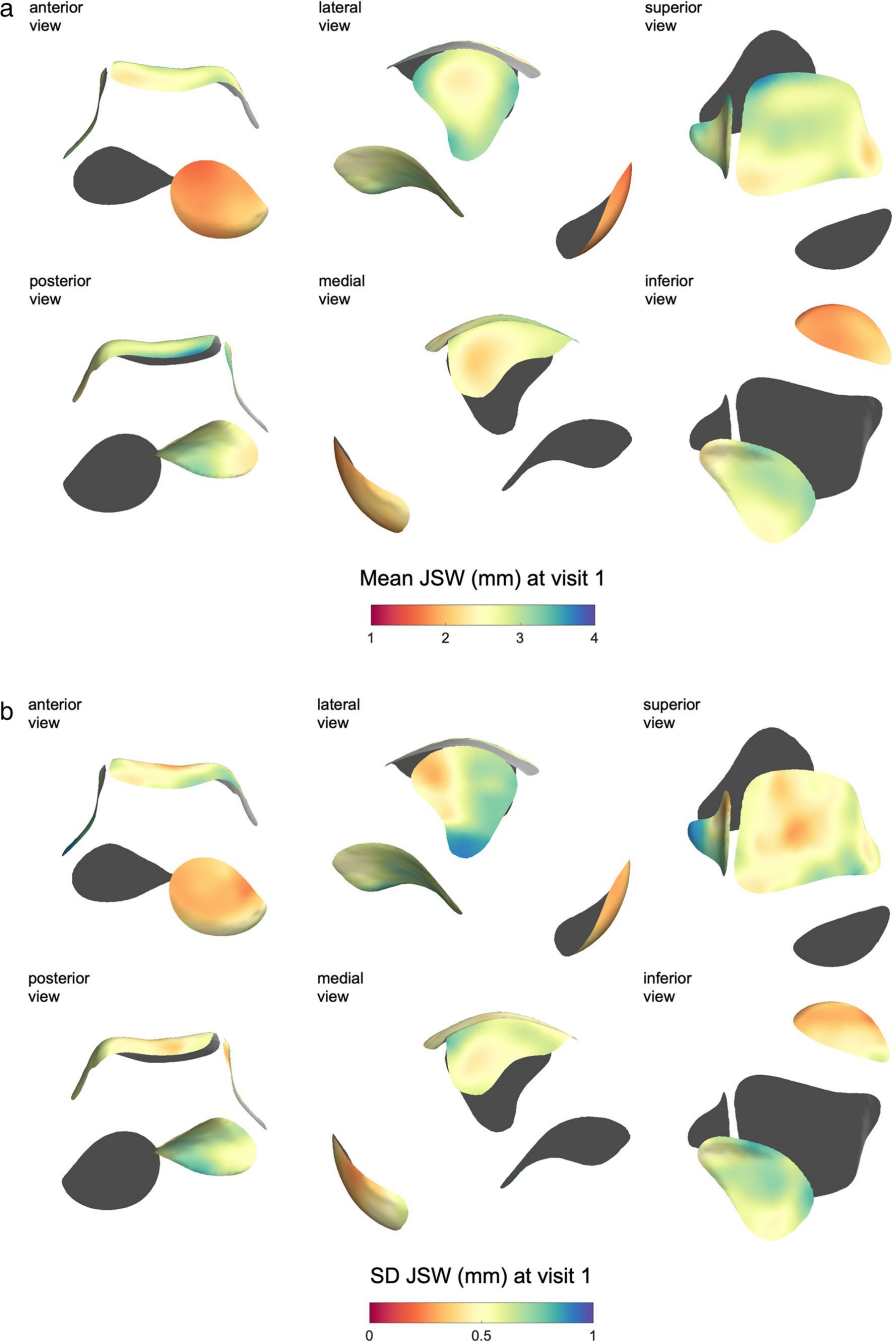
**a** Mean joint space width (JSW) values from the whole cohort displayed across the template. **b** Standard deviation (SD) of joint space width (JSW) values from the whole cohort displayed across the template. Inner surface aspects with respect to the talus are shaded grey

Mean and SD JSW distribution maps from visit 1 are shown in Fig. 5a and b. Patch average JSW ± SD values at visit 1 were talonavicular 1.91 ± 0.36 mm, dome-medial 2.73 ± 0.51 mm, lateral gutter 2.84 ± 0.62 mm, and posterior subtalar 2.65 ± 0.60 mm. The same measures at visit 2 were negligibly different: talonavicular 1.90 ± 0.38 mm, dome-medial 2.74 ± 0.54 mm, lateral gutter 2.86 ± 0.66 mm, and posterior subtalar 2.63 ± 0.63 mm. These results are presented in Table 3.

**Table 3:** Patch-averaged joint space width (JSW) ± standard deviation (SD) values from visit 1 and visit 2, bias and limits of agreement for inter-operator reproducibility, and test-retest repeatability at each of the four joint surfaces. For comparison, reconstructed isotropic voxel size was 0.37 mm

	JSW mean ± SD (mm)		Bias ± limits of agreement (mm)	
	Visit 1	Visit 2	Reproducibility	Repeatability
Talonavicular	1.91 ± 0.36	1.90 ± 0.38	− 0.01 ± 0.27	0.01 ± 0.26
Medial talocrural	2.73 ± 0.51	2.74 ± 0.54	0.05 ± 0.24	0.00 ± 0.28
Lateral talocrural	2.84 ± 0.62	2.86 ± 0.66	0.00 ± 0.37	− 0.02 ± 0.40
Posterior subtalar	2.65 ± 0.60	2.63 ± 0.63	− 0.01 ± 0.31	0.02 ± 0.34

### Reproducibility

**Fig. 6:**
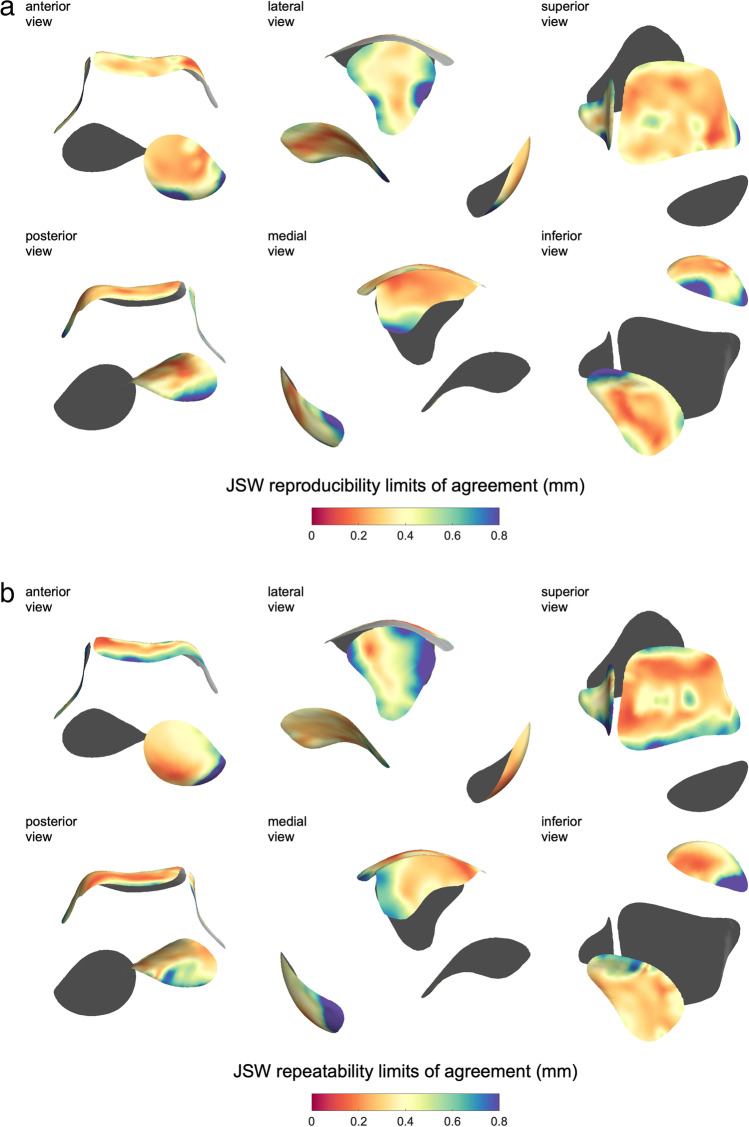
**a** Inter-operator joint space width (JSW) reproducibility limits of agreement (mm) displayed across the template. **b** JSW test-retest repeatability limits of agreement (mm) displayed across the template. Inner surface aspects with respect to the talus are shaded grey

Average bias for reproducibility was 0.00 mm when calculated across all joint facets. Patch average reproducibility bias ± LOA were talonavicular − 0.01 ± 0.27 mm, dome-medial 0.05 ± 0.24 mm, lateral gutter 0.00 ± 0.37 mm, and posterior subtalar − 0.01 ± 0.31 mm. Reproducibility bias results are presented as 3-D maps in Supplementary Fig. 5a, showing worst performance at the margins of the lateral gutter up to 0.3 mm in difference between operators. Reproducibility LOA results are presented as 3-D maps in Fig. 6a, with patch average values from each surface in Table 3.

### Test–retest repeatability

Average bias for test-retest repeatability was 0.00 mm when calculated across all joint facets. Patch average test-retest repeatability bias ± LOA were talonavicular 0.01 ± 0.26 mm, dome-medial 0.00 ± 0.28 mm, lateral talocrural − 0.02 ± 0.40 mm, and posterior subtalar 0.02 ± 0.34 mm. Repeatability bias results are presented as 3-D maps on the template in Supplementary Fig. 5b. In this case, we can see a similar surface variation as for reproducibility, but of a slightly smaller magnitude, apart from at the anterior margin of the lateral gutter where repeatability was worse than reproducibility. Repeatability LOA results are presented as 3-D maps in Fig. 6b, with patch average values from each surface in Table 3.

From Fig. 6b, optimum sensitivity (best test-retest repeatability) was less than ± 0.2 mm at each central joint surface. Worst repeatability (and reproducibility, Fig. 6a) performance was at the margins of the patches, with the lateral gutter, anterior talocrural joint, and anterior aspect of the posterior subtalar facet having a rim of values around ± 0.8 mm.

### Joint angulation

An increase in talocrural plantarflexion angulation by 1° was related to a significantly wider joint space by up to 0.1 mm at the anterior aspect of the lateral gutter, but also significantly narrower by up to ~ 0.05 mm at its posterior aspect. Further small regions of wider joint space by up to ~ 0.05 mm were noted at the anterior aspect of the talar dome (Supplementary Fig. 6).

## Discussion

Since 3-D imaging measurements at the foot and ankle have been shown to be fundamentally different but more reproducible and precise than 2-D measures [7], there still needs to be more research to establish the clinical value of 3-D approaches such as joint space mapping. As an important first step, our results show that it is possible to use joint space mapping to measure JSW in 3-D at multiple facets of the talus from WBCT imaging data. Although this has been achieved in several previous studies [4, 8, 9], we have for the first time analysed the inter-operator reproducibility and test-retest repeatability of 3-D JSW measurement at the ankle, demonstrating optimum limits of agreement nearly universally at values less than the isotropic imaging voxel dimensions of 0.37 mm. Repeatability limits of agreement equate to the smallest detectable difference that can be assumed to be not from factors related to imaging acquisition or measurement, and thus represent a sensitivity threshold above which any change in JSW upon repeat imaging can be assumed to be real [14]. As an example from our results, any difference between baseline and follow-up in average JSW at the talar dome and medial gutter greater than the threshold of 0.28 mm could be assumed to be real. Similarly, any average difference in dome-medial JSW recorded between operators greater than the threshold of 0.24 mm could be assumed real rather than from user factors.

The only result above this voxel dimension threshold was test-retest repeatability for the lateral gutter joint space, where the limits of agreement were ± 0.40 mm. This surface also demonstrated the worst inter-operator reproducibility bias (reaching at least 0.3 mm at its margins). The greatest standard deviation in JSW values from across the cohort was also seen at this surface (reaching up to 1 mm). This suggests that it is not only the most variable surface across the study population, but also most variable in how the joint perimeter is segmented by different users (from inter-operator reproducibility), and how it varies at follow-up when positioning is unconstrained (from test-retest repeatability).

We also noted that patch-averaged inter-operator reproducibility and test-retest repeatability error values were similar by location and varied in similar patterns between surfaces. In this study, we saw that the lateral gutter had greatest repeatability error (i.e. worst sensitivity), further explained by our 3-D angulation analysis showing that uncontrolled talocrural angulation was likely to be a contributing factor.

However, these patch average values do not represent the whole picture of JSW distribution and measurement performance: we have also demonstrated how JSW and error values vary across joint surfaces. This is an important ability when wanting to understand how different regions of a joint behave, particularly at the geometrically complex ankle. Our results show that poorest reproducibility and repeatability were found at the joint space margins (as seen before at the hip and knee [10, 12]), which skews a patch-averaged value away from the best performance usually seen at the central aspect of a joint surface. In this study, we saw that optimum sensitivity reached a smallest detectable difference of ± 0.15 mm at the central aspect of joint surfaces, meaning that JSM can be relied upon to detect meaningful differences in JSW between visits down to this threshold.

Our analysis has also given insight into the relationship between JSW distribution and talocrural joint angulation by taking advantage of the lack of controlled joint positioning between imaging visits. One prior study showed that mean compartmentalised tibiotalar JSW increased from dorsiflexion to plantarflexion [8]. Our study suggests that widening at the anteromedial talar dome and lateral gutter margins along with narrowing at the posterior aspect of the lateral gutter were significantly related to plantarflexion by up to 0.1 mm per degree of angulation difference. This could be explained by the shape of the talus where, during plantarflexion, the narrower posterior dome comes to lie more anteriorly in the mortise, thus increasing the joint space width.

We recognise that it is likely that 3-D measurements from WBCT imaging data will need to become automated in a ‘one touch’ process in order to become a clinically useful frontline diagnostic tool for clinicians [2]. Automatic segmentation methods already exist [18], but the challenge of automatically defining the margins of a joint surface remains unsolved. Although many studies have now used WBCT to investigate foot and ankle disorders, there is still a need to understand the implications for differences in imaging acquisition protocols, e.g. with respect to joint positioning and whether weight-bearing should be unilateral or bilateral [14]. Decisions on protocol standardisation would also need to ensure scanning times are tolerable for individuals with painful conditions so that they can maintain positioning without moving. An important part of evaluating WBCT imaging protocol suitability will be to know their reproducibility and repeatability. Although reproducibility is often cited in published studies, test-retest repeatability is rarely reported [14, 19], yet it is critical for understanding repeat performance. To the best of our knowledge, this has not been previously reported for 3-D JSW measurements at the ankle. Finally, there will be a need to prove the clinical value of measurements so that they are known to be useful to patients and clinicians for informing treatment choices and monitoring disease progression. This study represents a springboard for that next phase.

### Limitations

We recognise several limitations for this work. Prior to our analyses, there were initial technical exclusions because of motion (5 out of 30, 16.6%) and then subsequent measurement failures because of metal artefact (2 out of 25, 8%). This exemplifies the challenges in assessing foot and ankle orthopaedic patient populations such as this that can be expected to struggle to remain still and also have metalwork in situ. Metal artefact reduction software can be found in widespread clinical use and will be used in future analyses to see if this can be used to optimise joint space mapping in such circumstances [20]. We also recognise that we have assumed that there was no progression in joint space narrowing between imaging attendances, which was on average 74 days, but feel that this is a reasonable time period given that the joint space of a knee with osteoarthritis narrows radiographically by an average of 0.13 mm per year, equivalent to 0.03 mm over our same study interval [21]. Although not a direct comparison with values from the ankle, the sense of scale here suggests that this is an acceptable interval. A prospective study with set imaging intervals and a set positioning protocol would be valuable to investigate how to minimise test-retest repeatability error, but this study does at least represent performance in a realistic clinical setting.

## Conclusions

Results from this study indicate that optimum JSW mapping sensitivity in a standard foot and ankle orthopaedic clinical population reaches ± 0.15 mm, nearly half of the voxel dimension for the imaging system. Conversely, worst performance was seen at the lateral gutter (± 0.40 mm) where joint angulation is likely influencing JSW. Therefore, joint space mapping may have sensitivity to detect changes in 3-D JSW that may not be picked up by radiographs or other CT-based approaches, noting some limitations from the presence of metal-induced artefact that still need to be overcome. Having confidence in measuring JSW reliably should enhance our understanding of biomechanics at the ankle as well as provide more sensitive monitoring of joint space narrowing as a surrogate marker for progression in joint disease. Testing the clinical validity of joint space mapping in specific conditions will be important priorities for future research.

### Supplementary information

Below is the link to the electronic supplementary material.

Supplementary file1 (DOCX 47.3 MB)

## Abbreviations

JSM: Joint space mapping
JSW: Joint space width
LOA: Limits of agreement
SPM: Statistical parametric mapping
WBCT: Weight-bearing CT

## Declarations

### Guarantor

The scientific guarantor of this publication is Tom Turmezei.

### Statistics and biometry

Several of the authors have significant statistical expertise (Tom Turmezei, Graham Treece).

### Study subjects or cohorts overlap

N/A

### Methodology

• Retrospective

• Observational

• Performed at one institution
