# Supplementary material for: 3-D joint space mapping at the ankle from weight-bearing CT: reproducibility, repeatability, and challenges for standardisation
Source: Eur Radiol. 2023 May 31;33(11):8333–42. doi: 10.1007/s00330-023-09718-6 (PMC10598168; doi:10.1007/s00330-023-09718-6)
Supplement: Supplementary file 1 — Supplementary file1 (DOCX 47.3 MB) [file 330_2023_9718_MOESM1_ESM.docx]

**SUPPLEMENTARY MATERIAL**

**
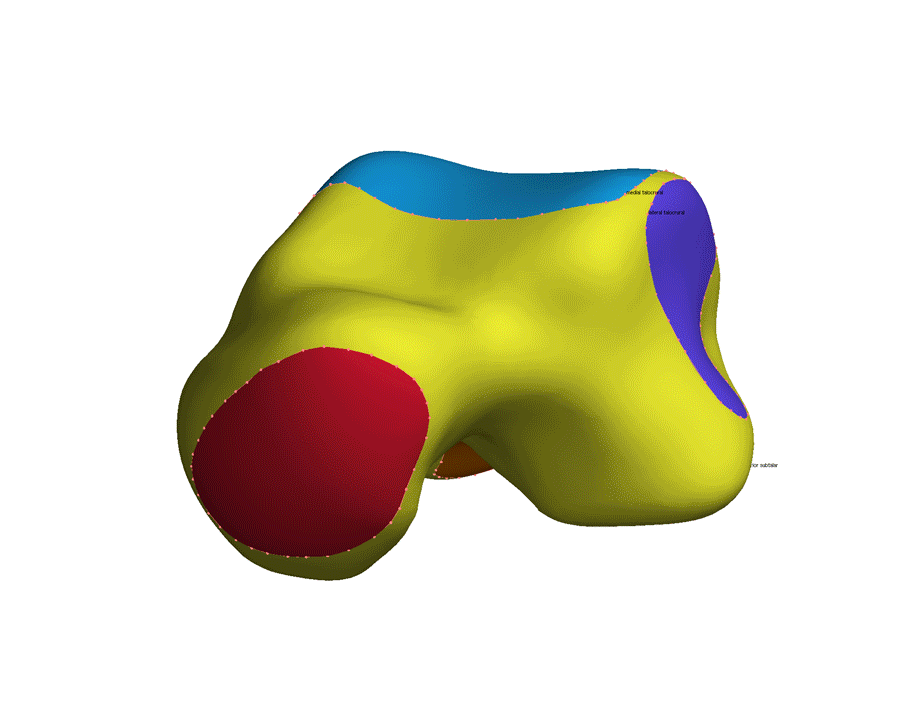
**

*Figure S1* Rotating image showing the four joint space patches extracted from the talus (yellow): medial talocrural (blue), lateral talocrural (purple), talonavicular (red), and posterior subtalar (orange).

*Figure S2* Rotating image showing the four joint space patches from SI Fig. 1 as a triangulated mesh rather than surface representation. Each vertex in a joint space patch mesh is a measurement point through the 3-D imaging data.

**
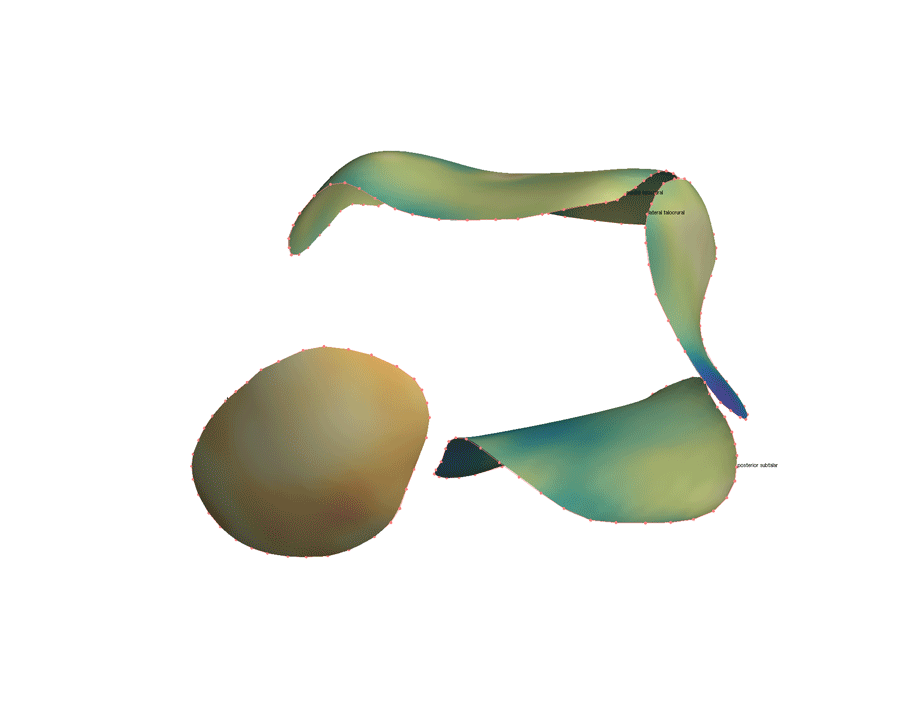
**

*Figure S3* Joint space width mapped out on each patch, with a relative colour scale: red = narrower, blue = wider.

*
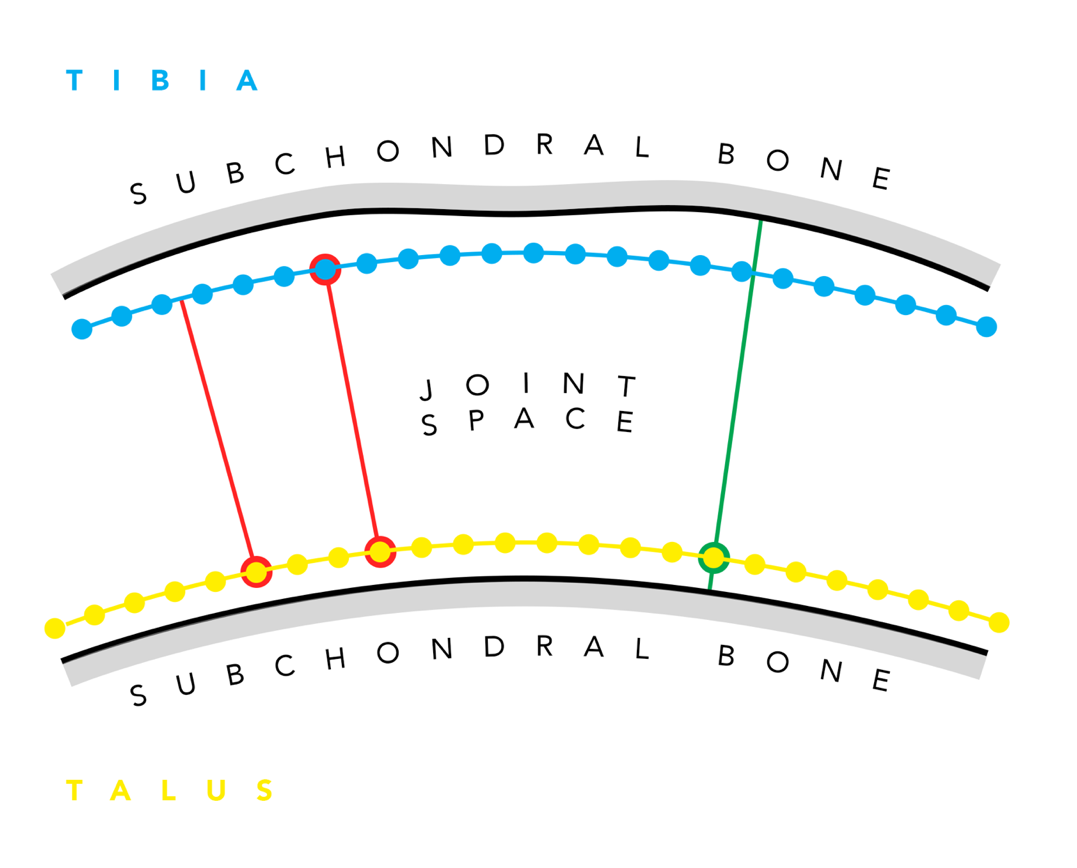
*

*Figure S4* 2-D graphical representation of 3-D JSM compared to mesh-to-mesh JSW measurement. The segmented talar bone mesh surface is shown as the yellow line with vertices in the mesh as dots. The tibial bone mesh surface across the joint space is shown in blue. Importantly, both are not precise representations of the real underlying articular subchondral bone surfaces, which are shown as the black lines. Mesh-to-mesh methods either take JSW as the distance between a vertex and the opposing mesh surface (left red dot and line) or as the closest vertex in the opposing mesh (right red dots and line). This will rely on the segmentation method and include any inaccuracies from this, often producing JSW values that are too narrow. JSM uses vertices in the talar mesh as a starting point to measure the distance between actual subchondral bone surfaces as defined by the optimiser algorithm (green dot and line), deblurring the imaging data to get a more accurate measurement of JSW.


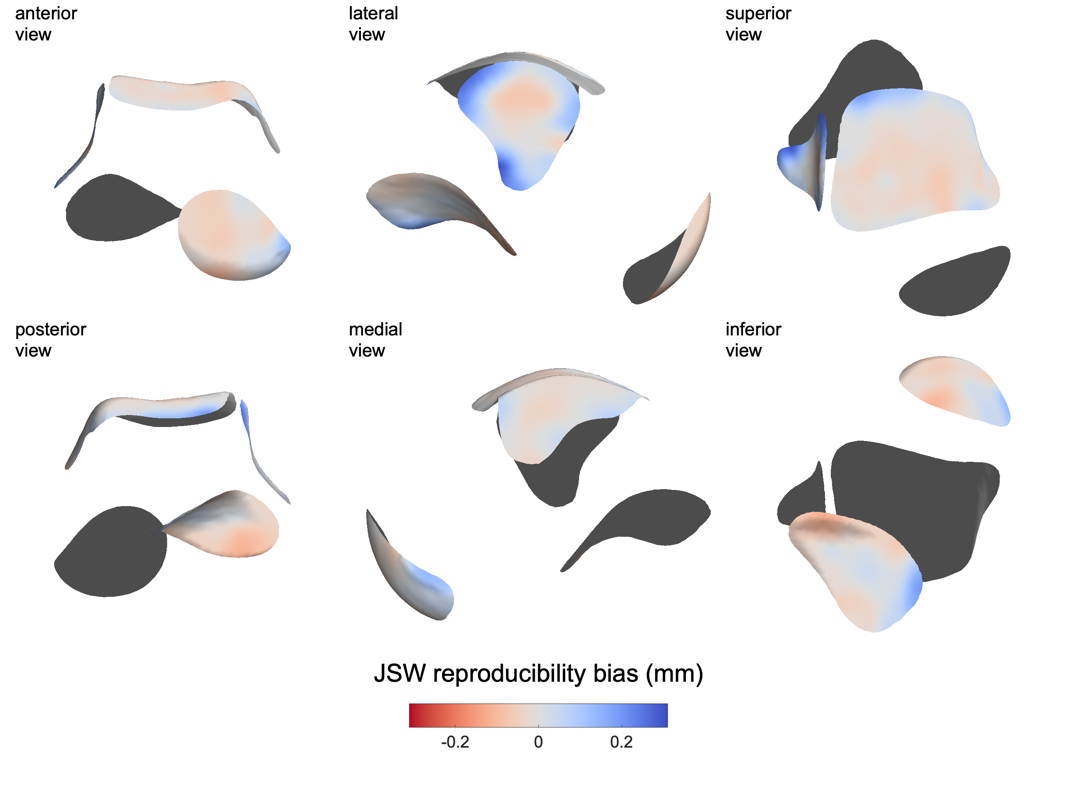


*Figure S5* (a) Inter-operator reproducibility bias in JSW distribution displayed across the template joint surfaces. Inner surfaces (with respect to the talus) are shaded grey.


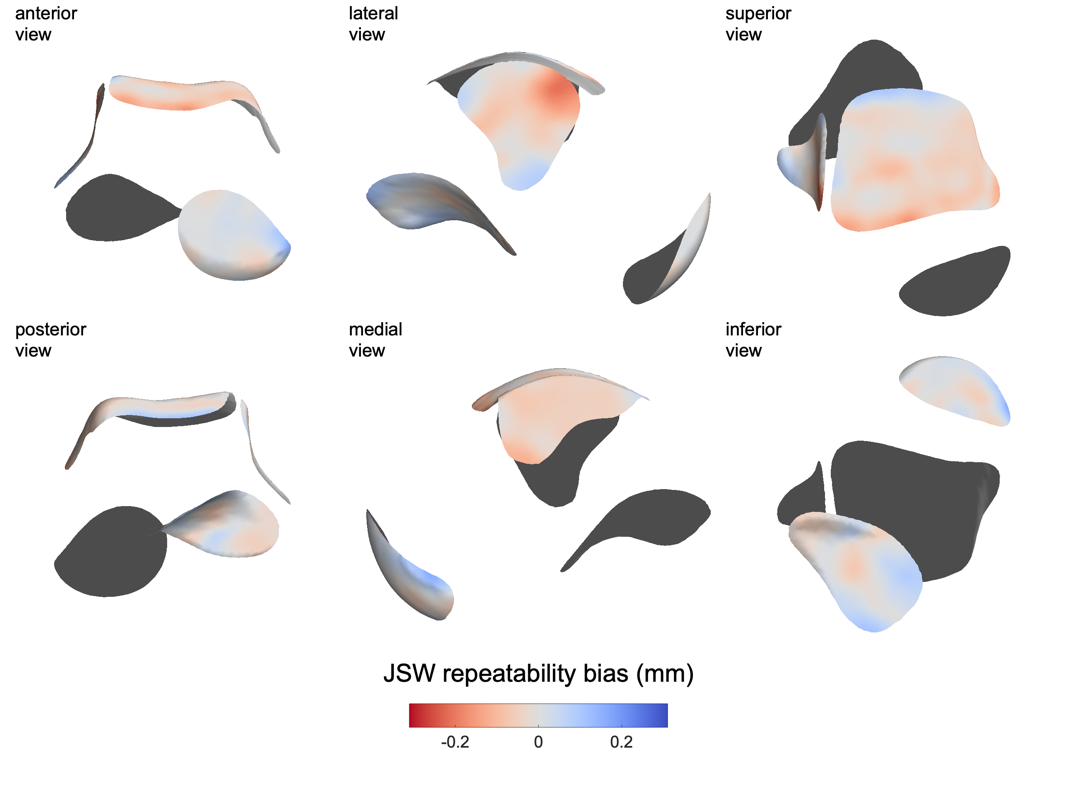


*Figure S5* (b) Test-retest repeatability bias in JSW distribution as visit 2 – visit 1 displayed across the template joint surfaces. Inner surfaces (with respect to the talus) are shaded grey.

*
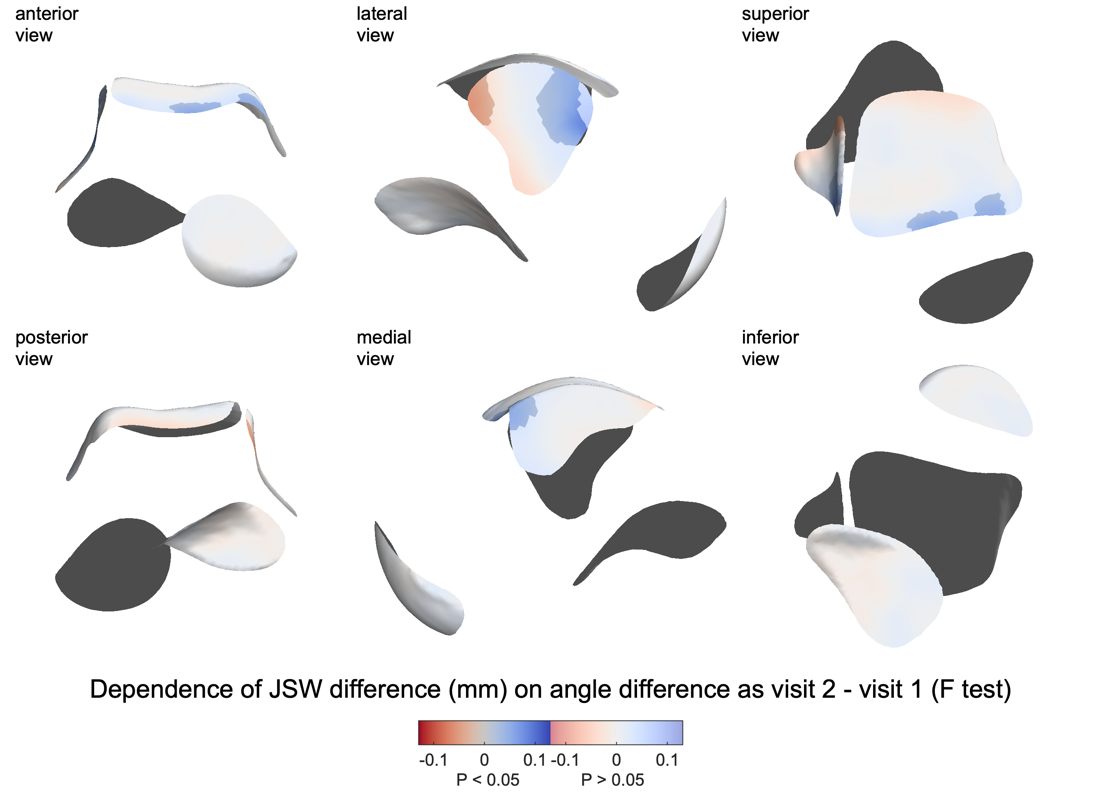
*

*Figure S6* The relationship between talocrural joint angulation and joint space width (JSW) as demonstrated with statistical parametric mapping. Significant regions (*P*<0.05) are shown as the unmasking of the colour map, e.g., at the anterior and posterior margins of the lateral talocrural joint patch (see lateral view) and across the anterior aspect of the talar dome (see superior view). The colour scale shows the amount of change in JSW for each degree increase in talocrural joint angulation (i.e., plantarflexion) as visit 2 - visit 1. Inner surface aspects with respect to the talus are shaded grey.
